# Dietary *β*-Hydroxy-*β*-Methylbutyrate Supplementation Affects Growth Performance, Digestion, TOR Pathway, and Muscle Quality in Kuruma Shrimp (*Marsupenaeus japonicas*) Fed a Low Protein Diet

**DOI:** 10.1155/2023/9889533

**Published:** 2023-01-20

**Authors:** Hua Mu, Chenbin Yang, Yu Zhang, Shengdi Chen, Panpan Wang, Binlun Yan, Qingqi Zhang, Chaoqing Wei, Huan Gao

**Affiliations:** ^1^Jiangsu Key Laboratory of Marine Bioresources and Environment, Jiangsu Key Laboratory of Marine Biotechnology, Jiangsu Ocean University, Lianyungang 222005, China; ^2^Co-Innovation Center of Jiangsu Marine Bio-Industry Technology, Jiangsu Ocean University, Lianyungang 222005, China; ^3^Marine Resource Development Institute of Jiangsu (Lianyungang), Lianyungang 222005, China; ^4^The Jiangsu Provincial Infrastructure for Conservation and Utilization of Agricultural Germplasm, Nanjing 210014, China; ^5^Ganyu Jiaxin Fishery Technical Development Co., Ltd., Lianyungang 222100, China

## Abstract

An 8-week feeding trial was performed to evaluate the effects of dietary *β*-hydroxy-*β*-methylbutyrate (HMB) supplementation on growth performance and muscle quality of kuruma shrimp (*Marsupenaeus japonicas*) (initial weight: 2.00 ± 0.01 g) fed a low protein diet. The positive control diet (HP) with 490 g/kg protein and negative control diet (LP) with 440 g/kg protein were formulated. Based on the LP, 0.25, 0.5, 1, 2 and 4 g/kg *β*-hydroxy-*β*-methylbutyrate calcium were supplemented to design the other five diets named as HMB0.25, HMB0.5, HMB1, HMB2 and HMB4, respectively. Results showed that compared with the shrimp fed LP, the HP, HMB1 and HMB2 groups had significantly higher weight gain and specific growth rate, while significantly lower feed conversion ratio (*p* < 0.05). Meanwhile, intestinal trypsin activity was significantly elevated in the above three groups than that of the LP group. Higher dietary protein level and HMB inclusion upregulated the expressions of target of rapamycin, ribosomal protein S6 kinase, phosphatidylinositol 3-kinase, and serine/threonine-protein kinase in shrimp muscle, accompanied by the increases in most muscle free amino acids contents. Supplementation of 2 g/kg HMB in a low protein diet improved muscle hardness and water holding capacity of shrimp. Total collagen content in shrimp muscle increased with increasing dietary HMB inclusion. Additionally, dietary inclusion of 2 g/kg HMB significantly elevated myofiber density and sarcomere length, while reduced myofiber diameter. In conclusion, supplementation of 1-2 g/kg HMB in a low protein diet improved the growth performance and muscle quality of kuruma shrimp, which may be ascribed to the increased trypsin activity and activated TOR pathway, as well as elevated muscle collagen content and changed myofiber morphology caused by dietary HMB.

## 1. Introduction

Aquaculture is the rapidly growing food production sector in the world, with the highest growth rate among animal production sectors [1]. About 70% of global aquaculture (excluding aquatic plants) is dependent on the supply of commercial compound feeds which account for more than half of the aquaculture production costs [2, 3]. Many aquatic animal species require a higher content of dietary protein to maintain the growth and health. The rapid and continuous expansion of world aquaculture has led to a heightened demand and a shortage of supply for protein sources, particularly for prime quality protein fishmeal. Thus, protein, as the most expensive nutrient in diet formulation, is crucial for the sustainable development of aquafeed and aquaculture [4]. Massive research efforts have been devoted in recent years to diminish the expense of feed and reliance on protein sources by reducing dietary protein or fishmeal level [5–7]. However, lower dietary protein or fishmeal level could exert negative impacts on growth, immunity, and muscle quality of farmed aquatic animals [8–11]. Therefore, except for developing novel protein sources, there is an urgent need to exploit safe and eco-friendly feed additives so as to ameliorate the adverse effects of low protein or fishmeal diets and sustain the global aquaculture.

It is well known that protein accretion is the primary determinant of body weight gain in aquatic animals [12]. The rate of protein deposition in the body is determined by the balance between protein synthesis and degradation in tissues (mainly muscle) [4]. Growth will occur when the rate of protein synthesis exceeds the rate of proteolysis in tissues, and protein synthesis, thus, is central to the growth of aquatic animals [13, 14]. Studies in mammals indicated that *β*-hydroxy-*β*-methylbutyrate (HMB), as a metabolite of leucine, is more potent than leucine in promoting protein synthesis and inhibiting protein degradation [15–17]. Dietary HMB supplementation has been found to improve the growth performance of Bama Xiang mini-pigs [18] and broiler chickens [19]. Meanwhile, dietary inclusion of HMB improved meat quality of Bama Xiang mini-pigs via manipulation of muscle fiber characteristics [20] and enhanced muscle mass by stimulating protein synthesis of pigs fed low protein diets [21]. Furthermore, the safety profile of HMB is quite unequivocal. For example, HMB has been popular with athletes and bodybuilders due to its function in increasing muscle strength and mass [22]. And plenty of studies showed that HMB supplementation or treatment is effective in preventing exercise-induced muscle damage in healthy subjects as well as muscle loss in pathological conditions characterized by high rates of muscle protein degradation [23–25]. What is more important, HMB is a nitrogen-free nutritional supplement, making the application of HMB in aquaculture eco-friendly.

Consistent with the findings in mammals, our unpublished data demonstrated that dietary HMB inclusion could also improve the growth performance and muscle quality of turbot (*Scophthalmus maximus* L.) and large yellow croaker (*Larimichthys crocea*), which may be imputable to the effects of HMB supplementation on protein metabolism and muscle fiber characteristics. However, the application efficacies of HMB supplementation in diets of other aquatic animal species (such as shrimp) are still unclear. Moreover, it is unknown whether dietary HMB effectively ameliorates the poor growth performance and muscle quality induced by lower dietary protein or fishmeal level in aquatic animals.

Kuruma shrimp (*Marsupenaeus japonicas*) is one of the most important cultured shrimps worldwide since its delicious taste, high market value, and excellent resistance to low temperature [26]. Aquaculture has become the best way to meet the market demand of this high-valued seafood due to its decreased wild catches [27, 28]. However, kuruma shrimp is commonly considered to have a higher requirement for dietary protein than other prawn species [29]. Reducing the finite and expensive protein in diet of kuruma shrimp has been shown to impair its growth performance [30]. Therefore, with the above in mind, the objective of this study was to evaluate the effects of dietary HMB supplementation on growth performance and muscle quality in kuruma shrimp fed with a low protein diet. The present finding would provide theoretical basis and guide for the application and research of HMB in prawn feed.

## 2. Materials and Methods

### 2.1. Ethics Statement

The experimental protocols and procedures for animal husbandry and handling were performed according to the Animal Care Committee of Jiangsu Ocean University in the present study.

### 2.2. Experimental Diets

Fishmeal, casein, soybean meal, and wheat gluten were used as the main protein sources, and fish oil and soybean lecithin were used as the main lipid sources to prepare the seven experimental diets. According to the dietary protein requirement (500 g/kg) reported by Teshima et al., [30] for kuruma shrimp, the positive control diet (HP) with 490 g/kg crude protein and negative control diet (LP) with 440 g/kg crude protein were formulated. Based on the LP, 0.25, 0.5, 1, 2, and 4 g/kg *β*-hydroxy-*β*-methylbutyrate calcium (HMB-Ca) (purity 97%, Shanghai Yuanye Bio-Technology Co., Ltd, China) were supplemented to design the other five isonitrogenous (440 g/kg protein) and isolipidic (75 g/kg lipid) diets which were named as HMB0.25, HMB0.5, HMB1, HMB2, and HMB4, respectively. The Table 1 shows the ingredients and proximate compositions, and Table 2 presents the amino acid composition of the experimental diets. Tryptophan was not determined due to the acid hydrolysis.

To prepare the experimental diets, all the dry ingredients were thoroughly crushed and passed through 80-mesh sieve and mixed fully by gradient dilution method according to the dietary formula. After that, fish oil with dissolved soybean lecithin and cholesterol was added and stirred thoroughly. Stiff dough was obtained by adding water and then extruded into pellets by the pellet-making machine (F-26 (II), South China University of Technology, China). Diets were finally dried in a ventilated oven at 40°C and stored at -20°C until use.

### 2.3. Growth Trial

The growth trial was carried out at the Jiangsu Key Laboratory of Marine Biotechnology, Jiangsu Ocean University, Lianyungang, China. Healthy kuruma shrimps were obtained from the Ganyu Jiaxin Fishery Technical Development Co., Ltd. (Lianyungang, Jiangsu Province, China). The shrimps were stocked in three circular tanks (500 L) and fed with the HP for 2 weeks to acclimate the laboratory conditions. After that, a total of 630 shrimp with homogenous size (2.00 ± 0.01 g) were randomly allocated into 21 rectangular polyvinyl chloride tanks (96 L, 30 shrimp per tank) corresponding to triplicate tanks of the seven dietary treatments. A net was used to cover each tank so as to prevent shrimp from jumping out, and sand (approximately 20 mm thickness) was distributed on the bottom of per tank. Shrimp were fed with the experimental diets at 4%-7% of the body weight for 56 days, and daily ration was divided into 30% at 08 : 00 and 70% at 20 : 00. Before the first feeding, about half of the water in each tank was replaced every second day. Weight gain (WG) and survival were determined every 2 weeks to adjust the feeding rate. Meanwhile, all the tanks including the sand were fully cleaned. During the feeding trial, the monitored water temperature, pH, salinity, dissolved oxygen and ammonia nitrogen were 25.0 ± 1.0°C, 8.1 ± 0.2, 28.0 ± 1.2, 6.1 ± 0.4 mg/L and <0.2 mg/L, respectively. Shrimps were cultured under natural light and dark regime.

### 2.4. Sample Collection

At the end of the growth trial, all shrimp were starved for 24 h. The total number and body weight of shrimp from each tank were recorded to calculate survival, WG, specific growth rate (SGR), and feed conversion ratio (FCR). Six shrimps from each tank were randomly obtained to collect the muscle and intestine samples for the analysis of proximate composition and digestive enzyme activity, respectively. Three shrimps per tank were randomly selected to obtain muscle samples for texture analysis. Another three shrimps from each tank were randomly picked to collect muscle for water holding capacity (WHC) and pH determination. Three muscles from each tank were randomly collected and stored at -80°C to determine the free amino acid (FAA) composition, contents of lactic acid, glycogen, total hydroxyproline (Hyp) and collagen, as well as the gene expressions. Three muscle samples (0.5 cm × 0.5 cm × 0.5 cm) from each tank were collected and soaked in 4% paraformaldehyde for paraffin section analysis. Another three muscles (0.2 cm × 0.2 cm × 0.2 cm) were sampled and kept in fixative solution (2.5% glutaraldehyde) for transmission electron microscopy analysis.

### 2.5. Analysis of Proximate Composition and Amino Acid in Diets and Muscle

The proximate analysis of diets and muscle was analyzed according to the standard methods [31]. Briefly, moisture content was measured by drying samples at 105°C until constant weight. The contents of crude protein and crude lipid in diets and muscle were determined by a Kjeldahl nitrogen analyzer (Kjeltec 8400, FOSS, Denmark) and a Soxhlet extractor (Soxtec 8000, FOSS, Denmark), respectively. Crude ash content of diets was measured by combusting samples in a muffle furnace at 550°C for 6 h. Amino acid profile in diets was analyzed using an automatic amino acid analyzer (L-8900, Hitachi, Japan) according to the method described by Qu et al. [32]. The FAA composition in muscle was detected by an automatic amino acid analyzer (L-8900, Hitachi, Japan) with a lithium high-performance column using the method of Xu et al. [33].

### 2.6. Digestive Enzyme Activity of the Intestine

Intestine samples of shrimp were weighed and diluted with normal saline at a ratio of 1 : 9, then homogenized on the ice and centrifuged for 10 min at 4°C (2500 r/min) to collect the supernatant for the analysis of digestive enzyme activities. The protein level in the obtained supernatant was measured using Coomassie brilliant blue (CBB) method. The activities of *α*-amylase (AMS), lipase (LPS), and trypsin (TRS) were detected using the commercial kits (Nanjing Jiancheng Bioengineering Institute, Nanjing, China).

### 2.7. Total RNA Extraction and Quantitative Real-Time Polymerase Chain Reaction

Total RNA was extracted from the shrimp muscle using total RNA isolation kit (Sangon Biotech (Shanghai) Co., Ltd., China). The integrity and concentration of RNA were detected by 1% agarose gel electrophoresis and GeneQuant pro (GE Pharmacia, USA), respectively. The isolated RNA was then reversely transcribed to cDNA by reverse transcription kit (Vazyme Biotech Co., Ltd., China).

The amplification reactions for quantitative real-time polymerase chain reaction (qRT-PCR) were performed in 96-well plates using a total volume of 20 *μ*L, containing 0.4 *μ*L of each primer, 4 *μ*L of cDNA template, 10 *μ*L of 2 × ChamQ SYBR qPCR Master Mix (Vazyme Biotech Co., Ltd., China), 0.4 *μ*L of ROX Reference Dye (Vazyme Biotech Co., Ltd., China), and 4.8 *μ*L of sterilized double-distilled water. The qRT-PCR was performed with an ABI StepOnePlus Real-Time PCR System using the following cycle conditions: 95°C for 30 s, followed by 40 cycles of 95°C for10 s, and 60°C for 30 s [34]. Melting curve analysis was carried out to validate that only one PCR product was obtained in these reactions. The expression levels of genes were quantified relative to the expression of *β*-actin using the comparative CT method (2^−△△CT^ method) [35]. The expression of *β*-actin was stable among the treatments. The gene expression was normalized with the LP group as control. The primer sequences of target genes (target of rapamycin (*tor*), ribosomal protein S6 kinase (*s6k*), eukaryotic translation initiation factor 4E (eIF4E)-binding protein 1 (*4e-bp1*), phosphatidylinositol 3-kinase (*pi3k*),and serine/threonine-protein kinase (*akt*)) were designed based on our transcriptome unigenes of kuruma shrimp (Table 3).

### 2.8. Muscle Texture Analysis, Water Holding Capacity (WHC) and pH Determination

The texture profile analyses (TPA) parameters (hardness, cohesiveness, springiness, and chewiness) were determined instrumentally by a texture analyzer (TMS-PRO, FTC, America) equipped with a 5 mm cylinder probe. The measurement conditions were set as double compression cycle test, constant speed of 30 mm/min, 60% deformation of the original thickness, and initial force of 0.1 N [36].

The muscle WHC was detected based on the gravimetric method [37]. In brief, 1 g of shrimp muscle was weighed (M) and wrapped with filter paper, then centrifuged at 4000 × g for 10 min at 4°C. The wet filter paper obtained by centrifugation was weighed (W1) and dried in the oven at 75°C to constant weight (W2). The WHC (%) was calculated as 100 − (W1–W2)/M × 100.

Three points in each muscle sample were selected to measure the pH values by using a portable pH meter (Testo 205, Testo AG, Lenzkirch, Germany).

### 2.9. Determination of Muscle Lactic Acid, Glycogen, Hydroxyproline and Collagen Contents

The contents of lactic acid, glycogen, and total Hyp in shrimp muscle were determined using the commercial kits (Nanjing Jiancheng Bioengineering Institute, Nanjing, China) following to the instructions. The total collagen content in muscle was calculated by multiplying the Hyp content by 8 [31] because Hyp accounts about 12.5% of collagen.

### 2.10. Muscle Paraffin Section Analysis and Transmission Electron Microscopy Analysis

Muscle paraffin section analysis and transmission electron microscopy analysis were performed according to the method described in a previous study [38]. Briefly, muscle samples (0.5 cm × 0.5 cm × 0.5 cm) were dehydrated with different concentrations of ethanol, embedded in paraffin wax, then sectioned, and stained with hematoxylin-eosin to observe the morphology. At least fifteen images from each muscle sample were captured by a microscope (Olympus, DP72, Tokyo, Japan). The ImageJ Launcher software was employed to count the myofiber number for calculating myofiber density and measure the myofiber diameter. Transmission electron microscopy analysis of muscle samples (0.2 cm × 0.2 cm × 0.2 cm) was carried out by Wuhan Google Biotechnology Co., Ltd. (Wuhan, China). Myofibrillar structure was observed to detect myofibrillar sarcomere length.

### 2.11. Statistical Analysis

All test data were analyzed using one-way analysis of variance (ANOVA) by the SPSS 17.0 software. The Tukey's test was used to assess the differences among seven treatment groups with the level of significance set at *p* < 0.05. Data were expressed as mean ± SE (standard error).

## 3. Results

### 3.1. Growth Performance

As shown in Table 4, no remarkable differences were observed in the initial body weight (IBW) and survival among the seven treatment groups (*p* > 0.05). After 8 weeks of feeding, the final body weight (FBW) of shrimp showed the highest value in the HP group (*p* < 0.05). Meanwhile, the shrimp fed HMB1 and HMB2 exhibited significantly higher FBW compared with the LP group. The HP group had significantly higher WG and SGR than those of the LP and HMB-supplemented groups (excluding HMB1), while no significant differences were found between the HP and HMB1 groups. The WG and SGR significantly increased in the HMB1 and HMB2 groups when compared with the LP group. The FCR in the HP, HMB1 and HMB2 groups was significantly lower than that in the LP group.

### 3.2. Muscle Proximate Analysis

Muscle proximate composition of kuruma shrimp fed with the seven experimental diets is presented in Table 5. Muscle moisture content in the HMB1 group was significantly lower than that in the HP and HMB2 groups. The shrimp fed HMB1 displayed significantly higher crude protein content in muscle when compared with the LP and HMB0.25 groups. The crude lipid content in muscle significantly decreased in the five groups of shrimp fed diets with HMB when compared with the HP and LP groups, with the lowest value in HMB2 group. However, there were no significant differences in contents of crude protein and crude lipid between the HP and LP groups.

### 3.3. Amino Acid Composition in Diets and Muscle

Nine essential amino acids (EAA) and eight nonessential amino acids (NEAA) were determined in the experimental diets (Table 2). The contents of most individual amino acids including leucine (Leu), total EAA, and NEAA in the HP diet were higher than those in the LP diet, while there were no obvious differences among the LP and HMB-supplemented diets.

The content of FAA in muscle of kuruma shrimp fed with the seven experimental diets is given in Table 6. Lower dietary protein level significantly reduced the contents of free threonine (Thr), Leu, valine (Val), phenylalanine (Phe), lysine (Lys), histidine (His), arginine (Arg), serine (Ser), proline (Pro) and glutamic acid (Glu), as well as the contents of total free EAA and total free amino acids (TAA) in shrimp muscle. However, compared with the LP group, the contents of above individual amino acid (excluding Ser), total EAA and TAA in muscle significantly enhanced in the HMB1 group, which were comparable with those of the HP group. Free Ser content in muscle of shrimp fed HMB2 and HMB4 was significantly higher than that in the LP group, although its value in HMB2 and HMB4 groups was still significantly lower than that of the HP group. Meanwhile, compared with the LP group, the HMB2 and HMB4 groups exhibited significantly higher contents of free Val, Phe, Arg, Pro and total EAA in muscle, which were at similar levels as the HP group. However, seven treatments showed no significant effect on the content of total delicious amino acids (glycine + alanine + aspartic acid + Glu) in shrimp muscle.

### 3.4. Digestive Enzyme Activity of the Intestine

**Figure 1:**
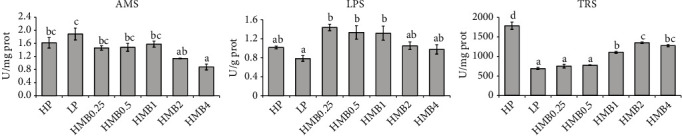
The activities of digestive enzymes in intestine of kuruma shrimp fed with the experimental diets. Values are expressed as the means ± SE (*n* = 3). Different lowercase letters above the bars present significant differences (*p* < 0.05). AMS: *α*-amylase; LPS: lipase; TRS: trypsin.

As shown in Figure 1, the AMS activity in intestine showed no significant difference among the HP, LP, HMB0.25, HMB0.5 and HMB1 groups. However, its activity in the shrimp fed LP was significantly higher than that in the HMB2 and HMB4 groups. The HMB0.25, HMB0.5 and HMB1 groups had significantly higher LPS activity in intestine than that of the LP group, while no significant difference was found between the HP and other six groups. Intestinal TRS activity showed the highest value in the HP group and the lowest value in the LP, HMB0.25 and HMB0.5 groups. However, compared with the LP, HMB0.25 and HMB0.5 groups, the HMB1, HMB2 and HMB4 groups had significant improvement in the TRS activity.

### 3.5. Expression Levels of TOR Pathway-Related Genes in Muscle

**Figure 2:**
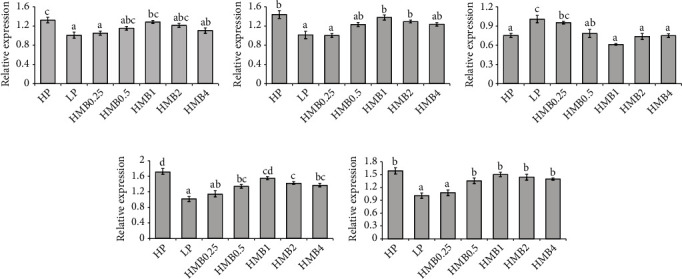
The relative mRNA expressions of *tor* (a), *s6k* (b), *4e-bp1* (c), *pi3k* (d), and *akt* (e) in muscle of kuruma shrimp fed with the experimental diets. Values are expressed as the means ± SE (*n* = 3). Different lowercase letters above the bars present significant differences (*p* < 0.05). *tor*: target of rapamycin; *s6k:* ribosomal protein S6 kinase; *4e-bp1:* eukaryotic translation initiation factor 4E (eIF4E)-binding protein 1; *pi3k*: phosphatidylinositol 3-kinase; *akt*: serine/threonine-protein kinase.

As displayed in Figure 2, the expression level of *tor* in muscle of shrimp fed LP and HMB0.25 was significantly lower than that in the HP and HMB1 groups. The HP, HMB1 and HMB2 groups had significantly higher expression level of *s6k* in muscle than that of the LP and HMB0.25 groups. The expression level of *4e-bp1* in muscle significantly increased in the LP group when compared with the HP and HMB-supplemented groups (excluding HMB0.25). The expression of *pi3k* in muscle of the HP and HMB-supplemented groups (excluding HMB0.25) was significantly higher than that of the LP group. Meanwhile, the LP and HMB0.25 groups had significantly lower expression level of *akt* in muscle than that of the other five groups, while no significant difference was found either between the LP and HMB0.25 groups or among the other five treatment groups.

### 3.6. Muscle Texture, Water Holding Capacity (WHC) and pH

As shown in Table 7, lower dietary protein level significantly decreased muscle hardness, springiness and chewiness. Compared with the LP group, shrimp fed HMB2 showed significant improvements in muscle hardness and chewiness. However, dietary HMB inclusion did not significantly affect muscle springiness. Meanwhile, all the dietary treatments had no significant effect on muscle cohesiveness. The muscle WHC significantly enhanced in the HMB2 group when compared with the LP, HMB0.25 and HMB0.5 groups. But it showed no significant difference between the HP and other six groups.

As shown in Table 8, there was no significant difference in muscle pH among the seven treatment groups.

### 3.7. Muscle Lactic Acid, Glycogen, Total Hydroxyproline and Collagen Contents

As displayed in Table 8, shrimp fed HMB1 and HMB2 exhibited a significant decrease in muscle lactic acid content compared with the LP group. The contents of total Hyp and collagen in muscle were significantly improved in HMB4 group compared with those in the LP and HMB0.25 groups. However, the contents of lactic acid, total Hyp and collagen in muscle showed no significant differences between the HP and other six groups. Meanwhile, there was no significant difference in muscle glycogen content among the seven treatment groups.

### 3.8. Morphology of Myofiber

**Figure 3:**
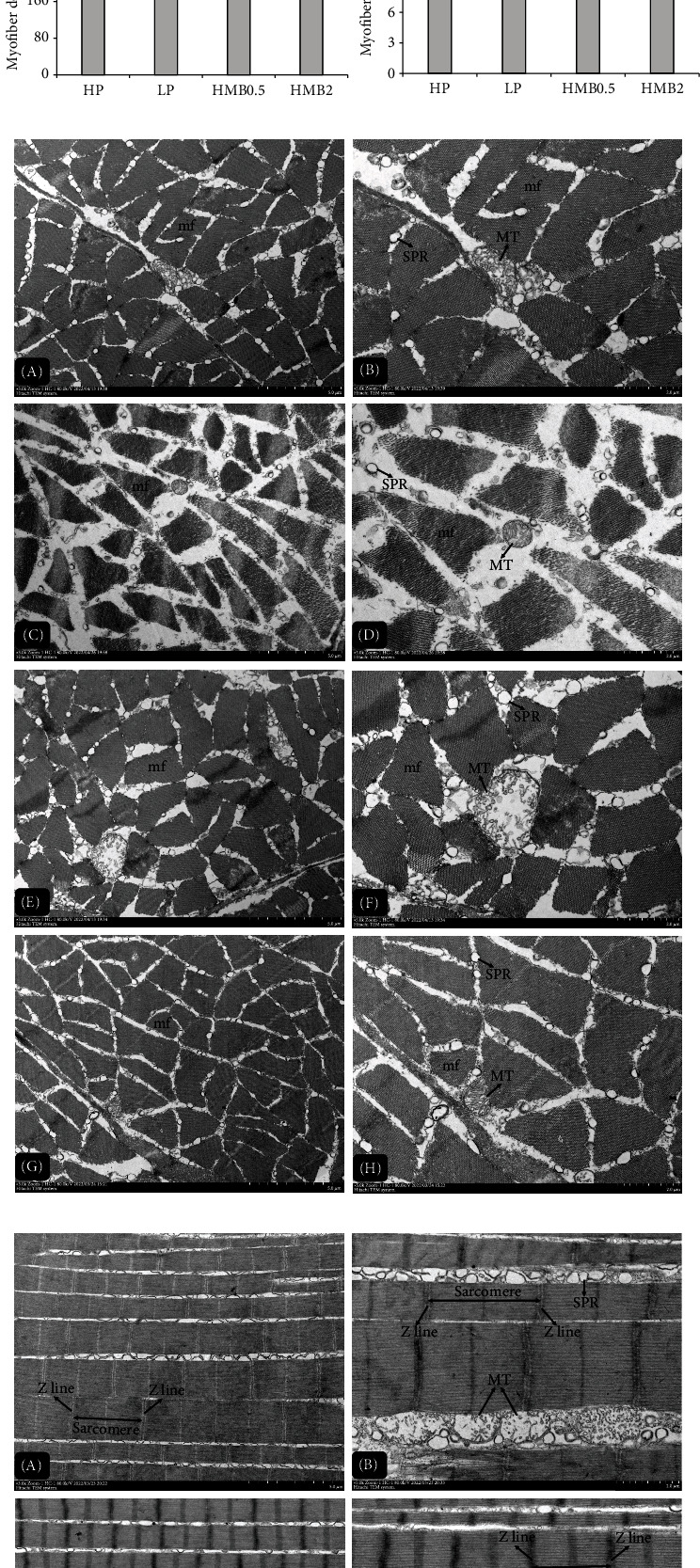
The morphology of myofiber in kuruma shrimp fed with the experimental diets. (a) Myofiber microstructure of cross sections. Magnification 200×. (b) Myofiber microstructure of longitudinal sections. Magnification 400×. (c) Myofiber density and diameter of kuruma shrimp. (d) Transmission electron micrographs of cross sections. (e) Transmission electron micrographs of longitudinal sections. (f) Myofibrillar sarcomere length of kuruma shrimp. A and B represent myofibrillar structure in the HP group taken at a magnification of 3000× and 5000×, respectively. C and D represent myofibrillar structure in the LP group taken at a magnification of 3000× and 5000×, respectively. E and F represent myofibrillar structure in the HMB0.5 group taken at a magnification of 3000× and 5000×, respectively. G and H represent myofibrillar structure in the HMB2 group taken at a magnification of 3000× and 5000×, respectively. MF: myofiber; FD: myofiber diameter; mf: myofibril; MT: mitochondrion; SPR: sarcoplasmic reticulum.

The Figure 3 shows the morphology of myofiber in kuruma shrimp fed with four experimental diets (HP, LP, HMB0.5 and HMB2). According to the results from myofiber microstructure of cross sections (Figure 3(a)), a tighter arrangement of myofiber was observed in shrimp fed HMB2. The myofiber microstructure of longitudinal sections (Figure 3(b)) showed that the HP and HMB2 groups had smaller fiber diameters, while fiber diameters of shrimp fed LP were widest. Based on the results from myofiber microstructure of cross and longitudinal sections, significant differences in myofiber density and diameter of shrimp were found among the four treatment groups (Figure 3(c)). The myofiber density significantly increased in the HMB2 group compared with that in the LP group, while there was no significant difference either among the HP, LP and HMB0.5 groups or among the HP, HMB0.5 and HMB2 groups. The HP, HMB0.5 and HMB2 groups had significantly smaller fiber diameter than that of the LP group. But it showed no significant difference among the HP, HMB0.5 and HMB2 groups.

Plentiful irregular myofibrils, complete and clear sarcomeres, sarcoplasmic reticulums, and mitochondria were observed in transmission electron micrographs of myofibrillar structure. The transmission electron micrographs of cross sections (Figure 3(d)) displayed that compared with the shrimp fed LP, the other three groups (especially the HP and HMB2 groups) had tighter myofibrillar structure and narrower intermyofibrillar spaces. From the transmission electron micrographs of longitudinal sections with the alternating dark, light areas and Z lines, the longest and shortest sarcomere length was found in the HP and LP groups, respectively (Figure 3(e)).  Figure 3(f) further indicated that sarcomere length in shrimp fed HP and HMB2 was significantly longer than that in the LP group, while no significant difference was observed either between the HP and HMB2 groups or between the LP and HMB0.5 groups.

## 4. Discussion

The rapid development of aquaculture has led to an increasing demand, depressed supply and high price of dietary protein sources. However, a high level of dietary protein has been generally recognized as necessary for good growth of kuruma shrimp due to its low utilization of dietary protein [30, 39]. In the present study, a low protein diet induced poor growth performance and feed utilization of kuruma shrimp, which is in agreement with previous studies in this species [30] and other shrimp [40, 41]. However, the present results suggested that supplementing 1 and 2 g/kg HMB in the low protein diet significantly improved the growth performance and feed utilization of kuruma shrimp. In particular, although the FBW of HP group was significantly higher than that of the other six groups, the shrimp fed a diet with 1 g/kg HMB had similar WG, SGR and FCR as the HP group. Similarly, our unpublished data indicated that 1 g/kg HMB inclusion in diet could also promote the growth performance of turbot and large yellow croaker. Meanwhile, dietary HMB has been proven to promote growth in Bama Xiang mini-pigs [18] and broiler chickens [19].

It was demonstrated that the growth of aquatic animals could be concerned with the digestive enzyme activity [42, 43]. In the present study, the shrimp fed with a low protein diet had significantly lower TRS activity in intestine, which may disturb the protein utilization and further cause the poor growth of shrimp. However, the activities of AMS and LPS in intestine were unchanged by the dietary protein levels, which may be ascribed to the similar levels of dietary carbohydrate and lipid [44]. All of the above observations about digestive enzymes were also demonstrated in oriental river prawn (*Macrobrachium nipponense*) fed different dietary protein levels [40]. Supplementation with 1, 2, and 4 g/kg HMB in the low protein diet significantly enhanced the intestinal TRS activity of shrimp. Meanwhile, the LPS activity in the shrimp fed a diet with 1 g/kg HMB was also significantly higher than that of the LP group. The increased activities of TRS and LPS possibly promoted digestion and absorption of nutrients and further growth of shrimp, as observed in the HMB1 and HMB2 groups (especially the HMB1 group). In ovo feeding, HMB has been found to increase jejunal nutrient uptake and digestion in turkeys [45]. However, there is no study performed to evaluate the impacts of dietary HMB on the digestion of aquatic animals. More studies are required to confirm the influence and mechanism of dietary HMB on digestion in various aquatic animals.

After nutrient such as protein is digested and resolved in the digestive tract, peptides and amino acids are absorbed by the intestine and then utilized for protein synthesis and other metabolism activities [46]. Protein synthesis generally determines the protein deposition, and the growth of aquatic animals is largely due to the protein deposition in tissues (mainly muscle) [12]. The TOR pathway is considered as a critical pathway which regulates the protein synthesis. Activated TOR stimulates translation initiation through activating s6k and inhibiting 4e-bp1 [47, 48]. The present data indicated that lower dietary protein level decreased the mRNA expression levels of *tor* and *s6k*, while enhanced the *4e-bp1* mRNA level in shrimp muscle, which may be related to the shortage of substrates (such as amino acids) for protein synthesis. The activation of the TOR pathway is largely regulated by the availability of amino acids [49, 50]; thus, the deficiency of some amino acids, especially EAA, could downregulate the TOR pathway [51, 52]. In the present study, the contents of most individual FAA, total EAA and TAA in muscle of shrimp fed LP were greatly lower than those of the HP group, which may induce the downregulation of the TOR pathway in the LP group. However, optimal dietary HMB inclusion increased the contents of most individual FAA, total EAA and TAA, and the mRNA expression levels of TOR and *s6k*, while reduced the *4e-bp1* mRNA level in muscle of shrimp fed with a low protein diet. These results suggested that supplementation with 1 g/kg HMB in a low protein diet could promote protein synthesis by upregulating the TOR pathway in shrimp muscle, which paralleled with the enhanced muscle protein content in the HMB1 group. This may contribute to the improved growth of the kuruma shrimp fed HMB1. A positive correlation between the WG and muscle protein content has been observed in abalone (*Haliotis discus hannai*) [43]. Similarly, HMB supplementation increased muscle mass via activating the TOR pathway in rats [53] and enhanced muscle protein synthesis by stimulating translation initiation in neonatal pigs [54]. It is well known that the TOR pathway is positively regulated by the PI3K/AKT pathway [55, 56]. The present data indicated that higher dietary protein level and dietary HMB supplementation both led to the upregulation of *pi3k* and *akt* mRNA levels in shrimp muscle, manifesting that the activated TOR pathway in the HP and HMB-supplemented groups may be partly explained by the upregulated *pi3k* and *akt* expressions. This is similar to what has been observed in previous studies, which found that HMB supplementation could result in the AKT activation in muscle of fasting rats [57] and enhance muscle mass by activating AKT signaling of pigs fed low protein diets [21]. Taken together, supplementation with 1 g/kg HMB in a low protein diet could promote muscle protein synthesis via activating the TOR related pathways, thus improving the growth of kuruma shrimp. However, the effect and underlying mechanism of dietary HMB on TOR-related pathways need to be thoroughly explored in more aquatic animals.

In addition to the growth performance, the muscle quality of aquatic animals is receiving the attention of researchers due to a growing global need for healthy, safe and nutritious high-quality meat. The texture parameters (hardness, cohesiveness, springiness, and chewiness) and WHC are some of the most considerable quality indicators, which determine consumer acceptance of aquatic animals [58, 59]. Generally, consumers show no preference for muscle with soft texture and poor WHC which could be improved by dietary nutrition [36, 38, 60]. In the current study, decreased muscle hardness, springiness and chewiness were found in the shrimp fed LP, denoting that lower dietary protein level could induce softer muscle texture in kuruma shrimp. However, we observed that improved muscle hardness, chewiness and WHC of shrimp were achieved at dietary HMB level of 2 g/kg, which is similar to our unpublished results in turbot and large yellow croaker. It has been demonstrated that muscle texture had a negative correlation with the muscle lipid content in aquatic animals [61, 62]. An interesting finding from the present study was that crude lipid content in shrimp muscle significantly decreased in response to dietary HMB supplementation, which may partially contribute to the improved muscle firmness of the HMB2 group. Previous studies reported that dietary HMB could decrease fat deposition in Bama Xiang mini-pigs [63] and broiler chickens [64] through regulating the *Bacteroidetes*-acetic acid-AMPK*α* axis and gut microbiota respectively, which needs to be further investigated and verified in aquatic animals.

Apart from the muscle lipid content, the muscle pH and collagen content were proven to influence muscle texture and WHC [65–68]. The muscle pH is mainly determined by the accumulation of muscle lactic acid which is generated by glycogen anaerobic breakdown [69, 70]. A lower muscle pH could decrease connective tissue strength, cause softer muscle texture and increase muscle liquid loss [68, 71]. However, the present data showed that muscle pH and glycogen content remained unaffected by dietary protein levels and HMB treatment, although 1 and 2 g/kg HMB supplementation significantly decreased lactic acid content in shrimp muscle. The total collagen content represented by the total Hyp concentration in muscle has been found to be positively correlated with the muscle hardness in large yellow croaker [36] and abalone [43]. Increased muscle collagen content was also demonstrated to improve muscle hardness in Pacific white shrimp (*Litopenaeus vannamei*) [38]. The current results indicated that the contents of total Hyp and collagen in shrimp muscle both increased with increasing dietary HMB inclusion, and the highest values were obtained in the 4 g/kg HMB group. The Hyp is produced by the hydroxylation of Pro and further used for the collagen biosynthesis [72]. It is interesting that HMB supplementation in the low protein diet also enhanced the muscle Pro content in the present study, which may contribute to the improvements in total Hyp and collagen contents, and texture and WHC of shrimp muscle. Further evidence was found in large yellow croaker, in which increased total Hyp and collagen contents in muscle were accompanied by elevated muscle hardness and lowered water loss in response to dietary Pro inclusion [73]. Taken as a whole, the beneficial effects induced by dietary HMB on muscle texture and WHC might be partially attributed to the enhancement of muscle collagen content in kuruma shrimp. However, the amounts of total Hyp and collagen in muscle were not significantly affected by dietary protein levels, although the shrimp fed HP exhibited relatively greater values.

Besides the abovementioned factors, it has been well-documented that muscle fiber characteristics are one of the pivotal factors affecting muscle texture and WHC [74, 75]. The higher myofiber density, smaller myofiber diameter, tighter myofibrillar arrangement, and longer sarcomere length could improve the muscle firmness and WHC of aquatic animals [38, 76]. Bearing in mind the observed impacts of dietary protein levels and HMB supplementation on muscle texture and WHC, it may be suggested that different dietary treatments affected muscle fiber characteristics in kuruma shrimp. To test this hypothesis, we then selected four treatment groups (HP, LP, HMB0.5 and HMB2) to observe the myofiber morphology of shrimp. As expected, lower dietary protein level decreased myofiber density and elevated myofiber diameter, while 2 g/kg HMB inclusion significantly enhanced myofiber density and reduced myofiber diameter in shrimp. These observations corresponded with the poor muscle texture and WHC of the LP group and improved muscle firmness and WHC of shrimp fed HMB2. In addition, ultrastructural examination revealed that the shrimp fed HP and HMB2 had obviously tighter myofibrillar arrangements, narrower intermyofibrillar spaces, and longer sarcomere lengths. The current results fit well with a previous study founding that dietary HMB could improve meat quality through increasing sarcomere lengths in pigs [20]. A longer sarcomere length could exert a beneficial effect on muscle quality via making a layer structure of muscle and preventing the soluble protein loss [77]. Overall, the low protein diet may induce poor muscle texture and WHC of kuruma shrimp by decreasing myofiber density and sarcomere length, as well as increasing myofiber diameter. Dietary HMB supplementation may improve muscle firmness and WHC via reducing myofiber diameter, elevating myofiber density and sarcomere length of kuruma shrimp.

## 5. Conclusion

In conclusion, the present study demonstrated that HMB could be regarded as a nutrition additive to improve the growth performance and muscle quality of kuruma shrimp fed with a low protein diet (440 g/kg protein), and the recommended level is 1-2 g/kg. The appropriate dietary HMB inclusion level increased the activities of digestive enzymes (TRS and LPS) in intestine and activated the TOR pathway in muscle, thus promoting the growth of shrimp. Meanwhile, optimal dietary HMB level improved muscle firmness and WHC of shrimp fed with a low protein diet, which may be ascribed to the lower muscle lipid content, higher muscle collagen content, reduced myofiber diameter, and increased myofiber density and sarcomere length caused by dietary HMB. Nevertheless, the findings from the current study are suggestive, and further exploration is warranted to validate them. Furthermore, more attention should be paid to the application efficacies of HMB supplementation in diets of other aquatic animal species.

## Figures and Tables

**Table 1:** Formulation and proximate composition of the experimental diets (g/kg dry matter).

Ingredients	HP	LP	HMB0.25	HMB0.5	HMB1	HMB2	HMB4
Fish meal	280	280	280	280	280	280	280
Soybean meal	200	200	200	200	200	200	200
Wheat meal	148.5	148.5	148.5	148.5	148.5	148.5	148.5
Wheat gluten	115	115	115	115	115	115	115
Casein	100	50	50	50	50	50	50
Squid meal	40	40	40	40	40	40	40
Microcrystalline cellulose	40	90	89.75	89.5	89	88	86
Fish oil	25	25	25	25	25	25	25
Soybean lecithin	10	10	10	10	10	10	10
Ca (H_2_PO_4_)_2_	15	15	15	15	15	15	15
Premix^a^	10	10	10	10	10	10	10
Cholesterol	5	5	5	5	5	5	5
Chloride choline	5	5	5	5	5	5	5
Attractant^b^	5	5	5	5	5	5	5
Calcium propionate	1	1	1	1	1	1	1
Tert-butylhydroquinone	0.5	0.5	0.5	0.5	0.5	0.5	0.5
HMB-Ca^c^	0.00	0.00	0.25	0.5	1	2	4
Total	1000	1000	1000	1000	1000	1000	1000
Proximate composition (g/kg)							
Moisture	84.2	74.9	85.7	77.5	74.1	78.3	72.6
Crude protein	488.3	440.1	438.7	436.2	436.6	439.3	439.8
Crude lipid	76.5	76.1	75.0	76.6	76.5	75.7	76.0
Ash	132.6	131.6	134.1	135.0	136.3	138.8	139.2

^a^ Warranty levels per kilogram of the product: retinyl acetate, 450,000 IU; cholecalciferol, 100,000 IU; D, L-*α*-tocopherol acetate, 5,000 mg; menadione, 500 mg; thiamine nitrate, 500 mg; riboflavin, 700 mg; pyridoxine hydrochloride, 600 mg; cyanocobalamin, 2 mg; calcium-D-pantothenate, 2,000 mg; niacin, 3500 mg; folic acid, 150 mg; D-biotin, 6 mg; ascorbic acid, 10,000 mg; inositol, 8,000 mg; magnesium, 20,000 mg; iron, 2,000 mg; zinc, 7500 mg; manganese, 2,000 mg; copper, 1500 mg; cobalt, 80 mg; selenium, 10 mg; iodine, 100 mg. ^b^ Attractant: glycine: betaine = 1 : 2. ^c^ HMB-Ca: *β*-hydroxy-*β*-methylbutyrate calcium.

**Table 2:** Amino acid composition of the experimental diets (% dry matter)^a^.

Amino acid	HP	LP	HMB0.25	HMB0.5	HMB1	HMB2	HMB4
Essential amino acids							
Threonine	1.38	1.16	1.16	1.21	1.16	1.17	1.13
Isoleucine	1.48	1.26	1.29	1.33	1.29	1.32	1.28
Leucine	2.89	2.46	2.49	2.57	2.49	2.54	2.46
Valine	1.69	1.41	1.43	1.49	1.43	1.46	1.42
Methionine	1.28	1.08	1.10	1.17	1.10	1.11	1.08
Phenylalanine	1.41	1.23	1.27	1.29	1.27	1.27	1.24
Lysine	2.07	1.77	1.85	1.93	1.86	1.87	1.80
Histidine	0.43	0.42	0.43	0.44	0.43	0.44	0.44
Arginine	1.66	1.51	1.57	1.58	1.53	1.54	1.55
*Σ*EAA^b^	14.29	12.31	12.59	13.01	12.57	12.72	12.39
Nonessential amino acids							
Tyrosine	0.21	0.20	0.18	0.20	0.18	0.19	0.19
Serine	1.67	1.39	1.34	1.39	1.34	1.36	1.30
Glycine	1.73	1.57	1.59	1.66	1.59	1.61	1.53
Alanine	1.65	1.45	1.47	1.55	1.48	1.50	1.43
Proline	2.45	2.23	2.33	2.47	2.35	2.39	2.25
Aspartic acid	2.82	2.46	2.46	2.58	2.48	2.51	2.41
Glutamic acid	7.54	6.39	6.25	6.37	6.21	6.33	6.17
Cysteine	1.35	1.22	1.19	1.24	1.22	1.26	1.20
*Σ*NEAA^c^	19.42	16.90	16.80	17.45	16.86	17.15	16.48
EAA/NEAA	0.74	0.73	0.75	0.75	0.75	0.74	0.75

^a^ Tryptophan was not determined due to the acid hydrolysis. ^b^*Σ*EAA: total essential amino acids. ^c^*Σ*NEAA: total nonessential amino acids.

**Table 3:** Primer sequences of target genes in real-time PCR analysis.

Target gene	Primer sequence forward (5′-3′)	Primer sequence reverse (5′-3′)
*tor*	GAGTTGAGGGAGGTGTCGTT	GGTTCTTGAGGTAGTTTGCA
*s6k*	CTCTGTCACACACACATTTTGC	CGATTGTCTTCTTCCTGTTCTC
*4e-bp1*	ACCTGAGAAGACTGAGCCT	GGAGAACTGGAGATGGAAA
*pi3k*	CTCTGTATCCATTCGCTTC	TGACTACTCCTCTCACCTCTT
*akt*	GTTGACTGGTGGGGTTATGGA	GGTGATGTAGAAGGGGTGATT
*β-actin*	AGTAGCCGCCCTGGTTGTAGAC	TTCTCCATGTCGTCCCAGT

Abbreviations: *tor*: target of rapamycin; *s6k*: ribosomal protein S6 kinase; *4e-bp1*: eukaryotic translation initiation factor 4E (eIF4E)-binding protein 1; *pi3k*: phosphatidylinositol 3-kinase; *akt*: serine/threonine-protein kinase.

**Table 4:** Growth performance of kuruma shrimp fed with the experimental diets.

Parameters	Diet treatments						
	HP	LP	HMB0.25	HMB0.5	HMB1	HMB2	HMB4
IBW (g)	2.02 ± 0.02	1.98 ± 0.00	2.03 ± 0.01	1.99 ± 0.01	1.99 ± 0.00	1.99 ± 0.03	2.00 ± 0.02
FBW (g)	4.55 ± 0.03^c^	3.92 ± 0.03^a^	4.09 ± 0.05^ab^	4.14 ± 0.09^ab^	4.26 ± 0.04^b^	4.21 ± 0.07^b^	4.12 ± 0.08^ab^
WG (%)^a^	125.13 ± 2.77^d^	98.14 ± 1.03^a^	101.85 ± 3.37^ab^	107.49 ± 3.13^abc^	114.43 ± 2.21^cd^	111.03 ± 1.52^bc^	106.16 ± 2.85^abc^
SGR (%/d)^b^	1.45 ± 0.02^d^	1.22 ± 0.01^a^	1.25 ± 0.03^ab^	1.30 ± 0.03^abc^	1.36 ± 0.02^cd^	1.33 ± 0.01^bc^	1.29 ± 0.02^abc^
FCR^c^	1.46 ± 0.02^a^	1.70 ± 0.01^d^	1.66 ± 0.04^cd^	1.60 ± 0.03^bcd^	1.54 ± 0.02^ab^	1.57 ± 0.01^abc^	1.62 ± 0.03^bcd^
Survival (%)^d^	85.56 ± 2.22	82.22 ± 1.11	86.67 ± 3.33	81.11 ± 2.22	83.33 ± 1.92	85.56 ± 2.94	83.33 ± 3.85

*Note:* Values are presented as the means ± SE (*n* = 3). Values not sharing a common superscript in the same row differ significantly (*p* < 0.05). Abbreviations: IBW: initial body weight (g); FBW: final body weight (g). ^a^ WG (weight gain, %) = (final body weight – initial body weight)/initial body weight × 100.^b^ SGR (specific growth rate, %/d) = (ln final body weight – ln initial body weight)/days × 100. ^c^ FCR (feed conversion ratio) = dry feed intake (g)/live weight gain (g).^d^ Survival (%) = (final number of shrimp/initial number of shrimp) × 100.

**Table 5:** Muscle proximate composition (% wet matter) of kuruma shrimp fed with the experimental diets.

Parameters	Diet treatments						
	HP	LP	HMB0.25	HMB0.5	HMB1	HMB2	HMB4
Moisture	77.39 ± 0.22^b^	77.04 ± 0.57^ab^	76.94 ± 0.16^ab^	76.61 ± 0.41^ab^	75.44 ± 0.46^a^	77.22 ± 0.19^b^	76.42 ± 0.02^ab^
Crude protein	20.59 ± 0.29^ab^	20.12 ± 0.64^a^	20.16 ± 0.04^a^	21.07 ± 0.49^ab^	21.98 ± 0.49^b^	20.41 ± 0.13^ab^	21.30 ± 0.00^ab^
Crude lipid	0.87 ± 0.00^e^	0.89 ± 0.01^e^	0.84 ± 0.01^d^	0.80 ± 0.00^c^	0.80 ± 0.00^c^	0.71 ± 0.00^a^	0.76 ± 0.00^b^

Note: Values are presented as the means ± SE (*n* = 3). Values not sharing a common superscript in the same row differ significantly (*p* < 0.05).

**Table 6:** Free amino acid composition (*μ*g/g) in muscle of kuruma shrimp fed with the experimental diets.

Amino acid	Diet treatments						
	HP	LP	HMB0.25	HMB0.5	HMB1	HMB2	HMB4
Threonine	119.74 ± 3.59^b^	69.09 ± 3.89^a^	82.43 ± 2.38^ab^	105.36 ± 5.90^ab^	112.73 ± 9.99^b^	107.62 ± 11.39^ab^	104.09 ± 12.49^ab^
Isoleucine	99.67 ± 4.79^cd^	75.78 ± 4.17^abc^	59.66 ± 4.80^a^	70.53 ± 5.71^ab^	110.57 ± 8.29^d^	98.59 ± 6.08^bcd^	91.66 ± 5.98^bcd^
Leucine	122.43 ± 5.43^b^	80.04 ± 5.80^a^	84.34 ± 4.23^a^	94.68 ± 8.60^ab^	130.00 ± 12.05^b^	113.56 ± 7.01^ab^	105.39 ± 7.70^ab^
Valine	352.45 ± 11.80^b^	250.68 ± 10.58^a^	260.77 ± 14.33^a^	331.24 ± 11.96^b^	336.46 ± 8.28^b^	329.75 ± 12.03^b^	344.68 ± 9.08^b^
Methionine	32.83 ± 3.66	22.39 ± 2.47	20.49 ± 2.20	21.78 ± 2.27	24.80 ± 2.50	23.44 ± 2.47	28.36 ± 2.67
Phenylalanine	111.16 ± 5.60^bc^	64.56 ± 3.59^a^	62.52 ± 3.60^a^	83.55 ± 4.45^ab^	115.65 ± 7.59^c^	106.31 ± 6.27^bc^	110.57 ± 7.79^bc^
Lysine	118.03 ± 7.12^c^	77.93 ± 5.39^a^	79.81 ± 5.96^ab^	82.40 ± 6.65^ab^	111.36 ± 7.92^bc^	121.83 ± 6.96^c^	108.92 ± 7.16^abc^
Histidine	141.65 ± 7.26^c^	83.85 ± 4.43^a^	85.79 ± 5.17^a^	106.33 ± 5.89^ab^	129.25 ± 6.39^bc^	106.62 ± 7.64^ab^	115.25 ± 8.89^abc^
Arginine	5958.37 ± 87.08^b^	4819.05 ± 174.02^a^	5253.47 ± 144.19^a^	5971.16 ± 203.27^b^	6231.11 ± 119.54^b^	6265.21 ± 145.05^b^	6109.81 ± 115.96^b^
Tyrosine	107.70 ± 6.77^abc^	74.71 ± 7.43^a^	72.64 ± 4.88^a^	94.48 ± 7.34^ab^	123.02 ± 6.18^bc^	129.52 ± 8.07^bc^	131.31 ± 10.49^c^
Serine	239.22 ± 10.03^d^	129.85 ± 5.97^a^	123.52 ± 8.82^a^	133.43 ± 8.80^ab^	157.86 ± 9.10^abc^	181.42 ± 13.30^bc^	188.72 ± 14.18^c^
Glycine	1544.81 ± 144.40	1374.38 ± 159.76	1502.33 ± 150.25	1563.50 ± 173.76	1655.83 ± 128.22	1564.86 ± 145.12	1548.25 ± 115.77
Alanine	5096.74 ± 173.15	4624.34 ± 117.79	4877.13 ± 146.85	5086.40 ± 130.40	5318.03 ± 204.55	4926.84 ± 139.64	4909.01 ± 155.87
Proline	3014.30 ± 211.99^d^	865.95 ± 176.77^a^	1577.00 ± 146.64^ab^	2100.63 ± 152.82^bc^	2295.96 ± 173.15^bcd^	2813.48 ± 157.53^cd^	2613.70 ± 116.10^cd^
Aspartic acid	286.97 ± 8.78	245.56 ± 17.39	235.68 ± 18.46	250.59 ± 11.61	236.74 ± 10.08	227.32 ± 14.41	221.16 ± 19.74
Glutamic acid	472.53 ± 14.59^d^	315.63 ± 19.95^a^	331.05 ± 15.60^ab^	385.78 ± 20.64^abc^	433.63 ± 17.41^cd^	407.17 ± 16.17^bcd^	356.97 ± 16.07^abc^
Cysteine	2185.99 ± 173.95	1890.70 ± 115.97	1905.86 ± 151.29	2297.90 ± 207.47	2248.51 ± 161.05	1968.55 ± 168.57	1927.66 ± 138.36
*Σ*EAA^a^	7056.32 ± 136.03^c^	5543.37 ± 213.75^a^	5989.29 ± 186.26^ab^	6867.03 ± 254.06^bc^	7301.92 ± 176.23^c^	7272.94 ± 194.20^c^	7118.73 ± 124.39^c^
*Σ*DAA^b^	7401.04 ± 340.58	6559.91 ± 310.26	6946.18 ± 328.07	7286.28 ± 336.25	7644.23 ± 359.10	7126.20 ± 312.43	7035.38 ± 298.72
*Σ*TAA^c^	20004.58 ± 849.67^b^	15064.49 ± 822.18^a^	16614.48 ± 808.16^ab^	18779.74 ± 951.70^ab^	19771.50 ± 873.74^b^	19492.09 ± 813.26^b^	19015.50 ± 671.79^ab^

Note: Values are presented as the means ± SE (*n* = 3). Values not sharing a common superscript in the same row differ significantly (*p* < 0.05). ^a^*Σ*EAA: total free essential amino acids. ^b^*Σ*DAA: total delicious amino acids (glycine + alanine + aspartic acid + glutamic acid). ^c^*Σ*TAA: total free amino acids.

**Table 7:** Muscle texture and water holding capacity of kuruma shrimp fed with the experimental diets.

Parameters	Diet treatments						
	HP	LP	HMB0.25	HMB0.5	HMB1	HMB2	HMB4
Hardness (N)	12.87 ± 0.66^b^	8.83 ± 0.28^a^	9.57 ± 1.33^ab^	10.13 ± 0.62^ab^	10.77 ± 1.09^ab^	12.83 ± 0.22^b^	12.28 ± 0.55^ab^
Cohesiveness	0.24 ± 0.03	0.17 ± 0.02	0.15 ± 0.01	0.17 ± 0.02	0.22 ± 0.03	0.17 ± 0.00	0.17 ± 0.01
Springiness (mm)	1.62 ± 0.06^b^	1.02 ± 0.08^a^	1.17 ± 0.14^ab^	1.26 ± 0.06^ab^	1.33 ± 0.15^ab^	1.45 ± 0.04^ab^	1.25 ± 0.15^ab^
Chewiness (mJ)	6.39 ± 0.78^c^	2.88 ± 0.38^a^	2.99 ± 0.33^a^	3.54 ± 0.53^ab^	4.33 ± 0.48^abc^	5.71 ± 0.18^bc^	4.30 ± 0.74^abc^
WHC (%)	90.97 ± 0.95^ab^	88.44 ± 0.50^a^	87.66 ± 0.44^a^	88.44 ± 0.78^a^	90.84 ± 1.41^ab^	92.82 ± 0.18^b^	91.29 ± 0.19^ab^

Note: Values are presented as the means ± SE (*n* = 3). Values not sharing a common superscript in the same row differ significantly (*p* < 0.05). Abbreviations: WHC: water holding capacity.

**Table 8:** The pH, contents of lactic acid, glycogen, total hydroxyproline and collagen in muscle of kuruma shrimp fed with the experimental diets.

Parameters	Diet treatments						
	HP	LP	HMB0.25	HMB0.5	HMB1	HMB2	HMB4
pH	6.90 ± 0.06	6.79 ± 0.08	6.87 ± 0.07	6.88 ± 0.12	6.98 ± 0.06	6.84 ± 0.05	6.92 ± 0.05
Lactic acid (mmol/g prot)	0.25 ± 0.03^abc^	0.39 ± 0.04^c^	0.35 ± 0.04^bc^	0.38 ± 0.01^c^	0.20 ± 0.02^a^	0.21 ± 0.01^ab^	0.34 ± 0.03^abc^
Glycogen (mg/g)	0.50 ± 0.00	0.57 ± 0.01	0.58 ± 0.02	0.55 ± 0.04	0.58 ± 0.02	0.60 ± 0.05	0.58 ± 0.04
Total Hyp (*μ*g/g)	82.37 ± 3.69^ab^	72.54 ± 3.27^a^	74.69 ± 5.64^a^	81.43 ± 5.76^ab^	88.96 ± 3.67^ab^	96.99 ± 6.58^ab^	103.04 ± 7.98^b^
Total collagen (*μ*g/g)	658.94 ± 29.50^ab^	580.31 ± 26.16^a^	597.54 ± 45.12^a^	651.44 ± 46.05^ab^	711.71 ± 29.35^ab^	775.95 ± 52.63^ab^	824.31 ± 63.81^b^

Note: Values are presented as the means ± SE (*n* = 3). Values not sharing a common superscript in the same row differ significantly (*p* < 0.05). Abbreviations: Hyp: hydroxyproline.

## Data Availability

The data that support the findings of this study are available from the corresponding authors upon reasonable request.
